# Foliar N Application on Tea Plant at Its Dormancy Stage Increases the N Concentration of Mature Leaves and Improves the Quality and Yield of Spring Tea

**DOI:** 10.3389/fpls.2021.753086

**Published:** 2021-10-15

**Authors:** Mei-Ya Liu, Dandan Tang, Yuanzhi Shi, Lifeng Ma, Qunfeng Zhang, Jianyun Ruan

**Affiliations:** ^1^Tea Research Institute, Chinese Academy of Agricultural Sciences, Hangzhou, China; ^2^Key Laboratory of Tea Plant Biology and Resources Utilization (Ministry of Agriculture and Rural Affairs), Hangzhou, China

**Keywords:** foliar N application, dormancy stage, total N, amino acid, polyphenol, tea plant

## Abstract

Over 30% of the Chinese tea plantation is supplied with excess fertilizer, especially nitrogen (N) fertilizer. Whether or not foliar N application on tea plants at the dormancy stage could improve the quality of spring tea and be a complementary strategy to reduce soil fertilization level remains unclear. In this study, the effects of foliar N application on tea plants were investigated by testing the types of fertilizers and their application times, and by applying foliar N under a reduced soil fertilization level using field and ^15^N-labeling pot experiments. Results showed that the foliar N application of amino acid liquid fertilizer two times at the winter dormancy stage was enough to significantly increase the N concentration of the mature leaves and improved the quality of spring tea. The foliar application of 2% urea or liquid amino acid fertilizer two times at the winter dormancy stage and two times at the spring dormancy stage showed the best performance in tea plants among the other foliar N fertilization methods, as it reduced the soil fertilization levels in tea plantations without decreasing the total N concentration of the mature leaves or deteriorating the quality of spring tea. Therefore, foliar N application on tea plants at its dormancy stage increases the N concentration of the mature leaves, improves the quality and yield of spring tea, and could be a complementary strategy to reduce soil fertilization levels.

## Introduction

Although plant leaf is specialized in capturing carbon dioxide (CO_2_) and light, its capacity to absorb nutrients has long been recognized and exploited in agriculture (Fernández and Eichert, 2009; Eichert and Fernández, 2012). Plant leaves absorb nutrients mainly through the stoma, cuticle hydrophilic pores, and plasmodesmata, and the absorption of nutrients determines the effectiveness of foliar fertilization (Eichert and Fernández, 2012). Foliar fertilizers are used when soil conditions limit the availability of soil-applied nutrients; this may occur if the rate of the loss of soil-applied nutrients is high or if the stage of plant growth, internal plant demand, and environmental conditions interact to limit the delivery of nutrients to critical plant organs (Fernández and Brown, 2013). Numerous studies have confirmed that foliar fertilization can help increase crop yield and improve crop quality (Fernández et al., 2009; Moreira et al., 2017; Li et al., 2018; Wang et al., 2019). Thus, the foliar application of fertilizers is of increasing importance in agricultural production worldwide.

Foliar application of nitrogen (N) is more efficient than the soil application of N, especially in terms of the percentage uptake of the applied N. Because urea has a lower salt index and is more rapidly absorbed into the leaf, it has been the most commonly and extensively used foliar N fertilizer (Gooding and Davies, 1992; Hasani et al., 2016). Currently, there has been an increasing interest in novel bio-organic liquid N fertilizers, especially those containing free amino acids hydrolyzed from plants or alga (Liu H. et al., 2016; Wang et al., 2019; Hassan et al., 2020). The ability of plants to acquire amino acids has been shown in both laboratory and field conditions, and amino acids are now treated as another source of N for plants (Adamczyk et al., 2020). The absorption of foliar N fertilizers by plants depends on several factors, such as the type and concentration of the foliar N fertilizers applied, the N or the nutrient status of both the plant and soil, the developmental stage of the plant, as well as environmental factors. Taking these into account, the appropriate timing and management of foliar N sprays have been well-developed to improve the efficacy of foliar N fertilization (Eichert and Fernández, 2012).

Tea plants (*Camellia sinensis* L.) are one of the most economically important crops in many tropical and subtropical countries (Chen et al., 2007; Wei et al., 2018). To obtain a high yield and quality of tea, a large number of chemical N fertilizers have been applied to the soil in tea plantations in China. Consequently, more than 30% of the Chinese tea plantations excessively apply chemical fertilizers (Ni et al., 2019). Excess N fertilizer application not only wastes energy but also causes environmental pollution, and has no beneficial effect on the quality of tea. Nowadays a major focus of research on tea plants is to develop a strategy that reduces the soil fertilization level and increases the quality and yield of tea.

Plant leaves have been reported as reserve organs that support shoot growth (Reader, 1978) and N is often stored in the leaves of evergreen plants (Babst and Coleman, 2018). In tea plants, more than 75% of the stored N within the whole plant is re-translocated to promote the sprouting of dormant buds (Okano et al., 1994). And 30% of this remobilized N is stored in the mature leaves (Okano et al., 1994). The quality of teas produced in early spring is largely dependent upon the remobilization of N reserves in the mature leaves (Liu et al., 2016). In a field experiment using the ^15^N-labeled urea as the foliar N applied to both surfaces of matured leaves before the sprouting of dormant buds, the amount of ^15^N in the shoots increased, whereas the ^15^N concentration in the mature leaves decreased with the growth of the young shoots (Fan et al., 2020). Recently, Ma et al. (2019) reported that tea plants can take up N at low winter temperatures under field conditions. The foliar spraying of N fertilizer usually occurs 2 weeks before the sprouting of dormant buds, or during the harvesting period of spring tea. However, it remains unclear whether the application of foliar N at the winter dormancy stage could increase the N levels in the mature leaves before the sprouting of dormant buds and improve the quality and yield of spring tea, and thus serve as an alternative or complementary strategy for reducing soil fertilization levels. Hence, the objective of this study was to investigate the effects of foliar N application on the canopy of tea plants during the dormancy period and whether this could be an effective strategy to reduce the soil fertilization levels in tea plantations.

## Materials and Methods

### Field Experiment 1

The effect of the foliar N application times on tea plants (*Camellia sinensis* L. cv. “Yabukita”) at the winter dormancy stage was tested in a 16-year-old commercial tea plantation owned by Shaoxing Royal Tea Village Co., Ltd., in Shaoxing in the Zhejiang province of China (120°69′E, 29°93′N). No specific permissions were needed for any of the locations or activities, and the field studies did not involve endangered nor protected species. The tea plants were fertilized by the owner of the plantation with pure N, phosphorus (P), and potassium (K) at the levels of 600, 232.5, and 248.9 kg⋅ha^–1^⋅year^–1^, respectively. The applied foliar N fertilizer was a commercially available amino acid liquid fertilizer (CLAA) (as described by Liu et al., 2016), containing 10% of soluble proteins and peptides and a free amino acid concentration of 124.16 g⋅L^–1^, and the concentration of the total N is 41.23 g⋅L^–1^. The composition of the free amino acid is as follows: (g⋅L^–1^): 15.5 Glu, 10.92 Asp, 10.5 Thr, 10.25 Arg, 9.45 Pro, 9.28 Leu, 7.11 Ala, 6.76 Tyr, 6.73 Ser, 6.25 Val, 6.2 Gly, 5.87 Lys, 5.68 His, 5.47 Phe, 4.47 Met, 2.02 Cys, and 1.68 g⋅Ile. In a given field, 17 continuous rows (1.8 m × 50 m for one row) of tea plants were selected from the beginning, the first row of tea plants beneath the road was set as a protection row, followed by three rows of each treatment with zero (T0; control), one (T1), two (T2), three (T3), and four (T4) times application of foliar N, and the last row within the 17 continuous rows was also designated as a protection row. As the user manual described, for 666.67 m^2^, one 500 ml bottle of the amino acid liquid fertilizer should be used. Thus, for each spraying, 202.5 ml liquid was diluted into 16 L water and sprayed on the canopy of the three rows of tea plants (270 m^2^). No burning symptoms were shown on the tea plants of each treatment. The first foliar N spray was applied on November 12, 2016, and the remaining foliar sprays were separated by a 2-week interval. To measure the total N concentration, the first mature leaves beneath the dormant buds were harvested on March 21, 2017. Samples were collected from the middle row of each treatment to avoid the effects of the fumes of the foliar N from the other treatments. The first sprouted young shoots with one bud and two leaves and were collected on April 13, 2017, to detect the total concentrations of the free amino acids and polyphenols. Additionally, the sprouting density, 100 buds weight, and yield of spring tea were recorded on April 13, 2017, using the quadrat (0.33 m × 0.33 m).

### Field Experiment 2

The field experiment to investigate the effect of different types of foliar N fertilizers on tea plants was conducted in a 20-year-old tea plantation owned by Shaoxing Yulong Tea Co., Ltd., in Shaoxing, Zhejiang China (120°61′E, 29°81′N). No specific permissions were needed for any of the locations or activities, and the field studies did not involve endangered nor protected species. The tea cultivar was also *Camellia sinensis* L. cv. “Yabukita.” The tea plants were fertilized by the owner of the plantation with pure N, P, and K at levels of 1,300, 174.6, and 290.4 kg⋅ha^–1^⋅year^––1^, respectively. The foliar N fertilizers tested in this experiment included 2% (w/v) urea (320 g urea dissolved in 16 L water) (T1), a CLAA (113.4 ml liquid diluted in 16 L water) (T2) (Liu et al., 2016; Jiangyin Lianye Bioscience and Biotechnology Co., Ltd.),^1^ and a commercially available auxin-containing foliar fertilizer widely used by the local tea farmers (one package dissolved in 16 L water) (T3) (Chayechayaduoduo from the Shouguang Lvyang Chemical Co., Ltd.).^2^ The concentrations of T2 and T3 were prepared according to their manuals which could be found on the websites of the companies mentioned above. All the concentrations were suitable for tea plants and no burning symptoms were shown. The foliar application of water was set as control (CK). For each treatment, four rows of tea plants were used (1.8 m × 28 m for one row as one biological replicate, three replicates included; the remaining one row was used to separate adjoining treatments). Based on the results of field experiment 1, foliar applications of N fertilizers on tea plants were performed two times at the winter dormancy stage (on December 1 and19, 2017) and two times at the spring dormancy stage (on March 6 and 13, 2018). The first mature leaves beneath the dormant buds were harvested on March 22, 2018, for the measurement of the total N concentration. The first sprouted young shoots with one bud and two leaves were collected on March 30, 2018 to detect the total concentrations of the free amino acids and polyphenols. The sprouting density, 100 buds weight, and yield of spring tea were calculated using the quadrat on March 30, 2018.

### Field Experiment 3

A field experiment investigating the effect of foliar N fertilization on tea plants grown in the presence of soil 25% lower than the local soil fertilization level was conducted in two different tea plantations. One was located in Shaoxing (same as that mentioned in field experiment 1), and the other in Zhouning in the Fujian province of China (119°38′E, 27°20′N). In Zhouning, the tea cultivar used was *Camellia sinensis* L. cv. “fuyun 6#,” and the tea field was a 30-year-old tea plantation. The foliar N fertilizer used for this experiment was the same as that used in field experiment 1, and the manner of foliar N application was the same as that mentioned in field experiment 2 (i.e., two times at the winter dormancy stage and two times at the spring dormancy stage). The canopy (4 × 1.8 m × 28 m = 201.6 m^2^) of tea plants were equally sprayed with 151.2 ml liquid amino acid fertilizer diluted in 16 L water. The samples collected from the tea plants grown under the local fertilization level were set as CK. The samples collected from the tea plants grown under the 25% reduced soil fertilization level with or without the application of foliar N fertilizer were designated as M+ and M−, respectively.

According to local practices, pure N, P, and K were applied at the rate of 900, 196.5, and 414.9 kg⋅ha^–1^⋅year^–1^, respectively, in Shaoxing; and at the rate of 500, 196.5, and 331.9 kg⋅ha^–1^⋅year^–1^, respectively, in Zhouning. Therefore, to fertilize tea plants at 25% lower levels, pure N, P, and K were applied at the rate of 675, 147.4, and 311.2 kg⋅ha^–1^⋅year^–1^, respectively, in Shaoxing; and at the rate of 375, 135.2, and 248.9 kg⋅ha^–1^⋅year^–1^, respectively, in Zhouning. The foliar spray was applied two times at the winter dormancy stage of 2016 (on November 11 and December 13 in Shaoxing; on December 6 and December 20 in Zhouning) and two times in the spring dormancy stage of 2017 (on February 15 and March 2 in Shaoxing; on January 16 and February 2 in Zhouning). To measure the total N concentration of the mature leaves, total amino acids and polyphenols concentrations of young shoots, sprouting density, 100 buds weight, and yield of spring tea, samplings were conducted at both tea plantations similar to those described above. In Shaoxing, all samplings were conducted on April 11, 2017. In Zhouning, the sampling for measuring the total N concentration of the mature leaves was carried out on March 7, 2017, while the samples for all other measurements were collected on March 23, 2017.

### Calculation of the Sprouting Density, 100 Buds Weight, and Yield of Spring Tea

A 0.33 m × 0.33 m large quadrat was applied to calculate the sprouting density, 100 buds weight, and yield of spring tea, and at least three boxes were used within one treatment. The sprouting density was calculated as the number of sprouted buds within one quadrat, and the 100 buds weight was determined by measuring the weight of one hundred buds within one quadrat. To measure the yield of the spring tea, young shoots with one bud and two leaves within one quadrat were collected and weighed, and then the yield was calculated as g⋅m^–2^.

### Pot Experiment 1

To further confirm the effect of the foliar N application on tea plants in winter dormancy, a pot experiment using ^15^N-labeling urea as the foliar N fertilizer was conducted at the Tea Research Institute, Chinese Academy of Agricultural Sciences (TRICAAS, 30°10′N, 120°5′E). Four rooted-cuttings of “longjing43” (*Camellia sinensis* L.) were planted in one plastic pot containing 20 kg of commercial growth medium consisting of perlite, vermiculite, and peat. The pots were placed out in the open under full sunlight and were watered regularly. There were four treatments of foliar ^15^N-urea application, two times at the winter dormancy stage (TW) (on November 29 and December 17, 2018), two times in spring before the sprouting of dormant buds (TS) (this is also the dormancy stage of tea plants, so we presented it as the spring dormancy stage in the following text) (on February 14 and 28, 2019), a combination of two times at the winter and spring dormancy stage (TWS) (the timing was the same as that in TW and TS), and no ^15^N-urea application (CK). In each treatment, five pots of tea plants were used, and 2 L of 2% ^15^N-urea [40 g of the commercial ^15^N-urea (^15^N enrichment 5.16%) dissolved in 2 L of water, with an N concentration of 9.2 g⋅L^–1^] was sprayed evenly on the canopy of the tea plants. When spraying, the surface of the soil was covered with plastic to avoid the foliar N leaching into the soil of each pot. On March 28, 2019, all of the first sprouted young shoots with one bud and two leaves from the same pot were collected together for the determination of the total N or ^15^N concentration, total amino acids, and polyphenols, as well as the calculation of the 100 buds weight. The first mature leaves beneath the sprouted shoots were collected for the measurement of the total N or ^15^N concentration at the same time.

### Pot Experiment 2

The effect of foliar N application on the tea plants under reduced soil fertilization was also investigated using the ^15^N-labeled urea in a pot experiment at TRICAAS. Four rooted-cuttings of “longjing43” were planted in one plastic pot containing 20 kg of commercial growth medium consisting of perlite, vermiculite, and peat. Two levels of soil N fertilizer input were tested: 2 g of pure N per pot, which mimicked the normal fertilization level of 450 kg⋅ha^–1^ of pure N in the tea plantation (T2), and 25% reduced input of soil N fertilizer (i.e., 1.5 g of pure N per pot; T1). The tea cuttings grown under these two N levels were sprayed with or without foliar ^15^N-urea. The spraying of foliar ^15^N-urea was performed as described in pot experiment 1, and five pots were used per treatment.

### Determination of Total N, Free Amino Acids, Polyphenols, and ^15^N Concentrations

All the harvested mature leaves and young shoots were heated in a microwave oven for 2 min to deactivate the polyphenol oxidase and then dried in an electric oven at 60°C to a constant weight. The dried samples were ground into a fine powder using a ball mill (Mixer Mill MM300; Retsch, Germany). To measure the total N concentration of the mature leaves, 0.1 g of each dried sample was examined in an elemental analyzer (Vario Max CN Analyzer; Elementar Analysensysteme GmbH, Germany). The concentrations of the total free amino acids and total polyphenols in the young shoots (0.1 g dried sample) were extracted in 5 ml boiling water for 5 min. The total free amino acids concentration was measured using the ninhydrin method with glutamic acid as a standard, while the total polyphenols were measured using the Folin-Ciocalteu method with gallic acid as the standard (Ruan et al., 2010). The abundance of ^15^N in each sample was measured automatically with the Flash 2000 HT/MT253 Elemental Analyzer (EA)-Isotope Ratio Mass Spectrometer (IRMS) (Thermo Fisher Scientific, Waltham, MA, United States). The ^15^N derived from the ^15^N-urea application (N_dff_) was calculated as described by Fan et al. (2020).

### Statistical Analysis

The significance of the different treatments within one experiment was evaluated using one-way ANOVA followed by the Duncan test at a significance level of *P*_0_._05_ in the SPSS 22.0 software (IBM, Armonk, NY, United States).

## Results

### Effect of Foliar N Application Times on Tea Plants at Winter Dormancy Stage

**FIGURE 1:**
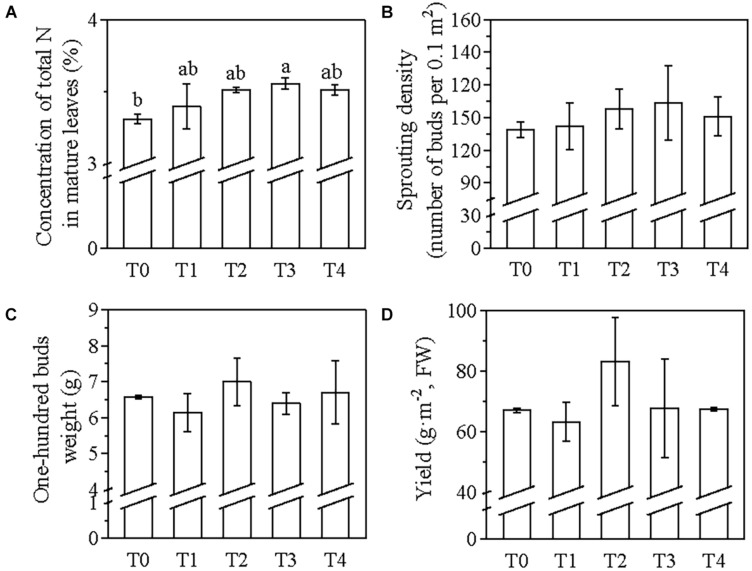
The effect of foliar N application times at the winter dormancy stage of tea plants on the total N concentration of the mature leaves **(A)**, sprouting density **(B)**, 100 buds weight **(C)**, and yield of spring tea **(D)**. A commercially available amino acid liquid fertilizer was applied to the tea plants one time (T1), two times (T2), three times (T3), or four times (T4); no foliar application of N (T0) was used as a control. The values were shown as means ± standard errors (SEs) (*n* = 3 or 4). The significance of the different foliar N application times was evaluated using one-way ANOVA followed by a Duncan test at a significance level of *P*_0_._05_ in the SPSS 22.0 software. Statistically significant differences (*P* < 0.05) among treatments were shown with a lower case letter displayed above the bar plot. There was no significant difference for sprouting density **(B)**, 100 buds weight **(C)**, and yield **(D)** of spring tea under different application times of foliar N at the winter dormancy stage. FW, fresh weight.

After the application of the foliar N fertilizer, the total N concentration of the mature leaves in the T3 treatment was significantly higher than that of T0, increasing by 7.5% (Figure 1A). No significant difference was detected among the other treatments (Figure 1A). The total N concentration of the tea plants in T2, T3, and T4 showed no significant difference compared with T1 (Figure 1A). The changes in the sprouting density among the treatments did not show a significant difference, but the increment in T3 and T2 was higher than that of T1 and T4 compared with T0 (Figure 1B). Additionally, compared with T0, the 100 buds weight showed the highest increase in T2, followed by T4 (Figure 1C). By contrast, in the T1 and T3 treatments, there was a decline in the 100 buds weight, compared with T0 (Figure 1C). Moreover, only the yield of the spring tea in T2 was higher than that of T0.

**FIGURE 2:**
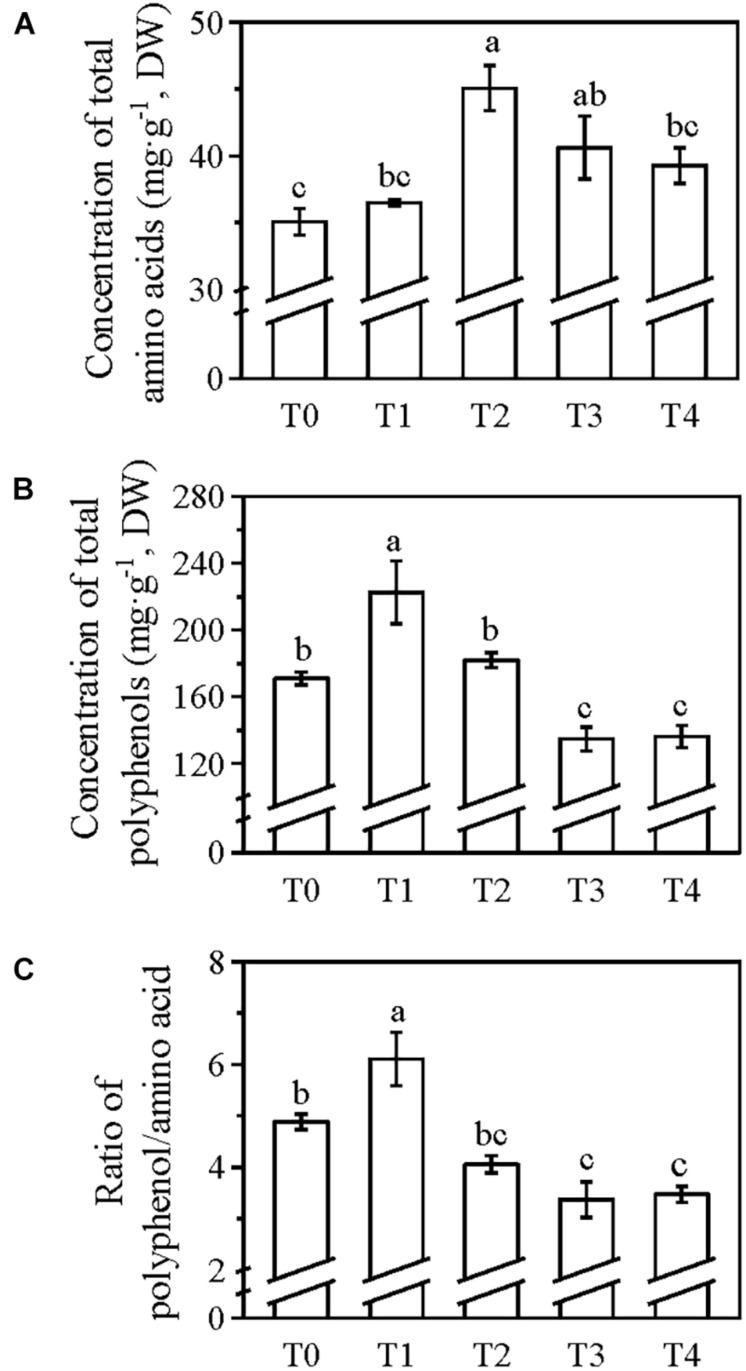
Concentrations of total amino acids **(A)**, polyphenols **(B)**, and the polyphenol/amino acid ratio **(C)** of spring tea from tea plants grown under different application times of foliar N in winter dormancy. A commercially available amino acid liquid fertilizer was applied to the tea plants at T1, T2, T3, or T4; no foliar application of N (T0) was used as control. Values were shown as means ± SEs (*n* = 4). Lowercase letters indicated the significant differences (*P* < 0.05; ANOVA, followed by the Duncan test). DW, dry weight.

Although no significant difference was detected in the sprouting density, 100 buds weight, and yield of spring tea among the five treatments (Figures 1B–D), the quality-related components of spring tea varied significantly among these treatments (Figure 2). The umami taste is believed to be mainly contributed by the free amino acids, while polyphenols have been reported to impose the astringent taste (Dai et al., 2015). Good quality green tea has a relatively higher level of free amino acids and lower concentration of polyphenols, and thus a low polyphenol/amino acid ratio. After the application of foliar N, the total concentration of amino acids increased by 4% in T1, 28.5% in T2, 15.9% in T3, and 12% in T4 compared with the T0 treatment (Figure 2A). Compared with T0, the total concentration of polyphenols decreased by 21% in T3 and 20% in T4 and increased by 30% in T1 and 6.5% in T2 (Figure 2B). The polyphenol/amino acid ratio in T1 was the highest among all treatments (6.11) including T0 (Figure 2C); this ratio was lower in T2, T3, and T4 compared with T0, although no significant difference was observed within the three treatments (Figure 2C). Therefore, the application of foliar N two times at the winter dormancy stage was sufficient for increasing the N concentration of the mature leaves and for improving the quality of the spring tea. In the following experiments, foliar N fertilizer was applied two times to tea plants at the winter dormancy stage.

### Confirmation of the Frequency of Foliar N Application Using the ^15^N-Urea

**FIGURE 3:**
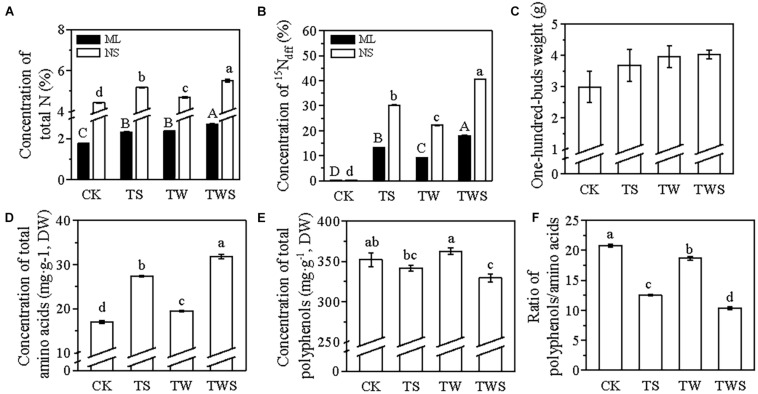
^15^N-labeling pot experiment investigating the effects of foliar N application on tea plants at spring dormancy stage, winter dormancy stage, and both the spring and winter dormancy stage. ^15^N-urea (2%, w/v) was sprayed on the tea plants two times at the winter dormancy stage (TW), two times at the spring dormancy stage (TS), or two times each at the winter or spring dormancy stage (TWS), no application of ^15^N-urea was used as control (CK). ML, mature leaves (black bars); NS, new shoots (white bars); ^15^N_dff_, ^15^N derived from the foliar ^15^N-urea; DW, dry weight. Data represented mean ± SE; *n* = 3 in panels **(A,B)**, 5 in panel **(C)**, 9 in panel **(D)**, 6 in panel **(E)**, and 3 in panel **(F)**. Lowercase letters indicated the significant differences of NS, while capital letters presented the significance of ML (*P* < 0.05; ANOVA, followed by the Duncan test).

The changes in the total N concentration and ^15^N_dff_ in the mature leaves and new shoots showed the same pattern with each other (Figures 3A,B). The ^15^N concentration of the mature leaves and new shoots of tea plants in the TWS treatment was significantly higher than in the other three treatments (Figures 3A,B). The foliar spray of N on tea plants increased the 100 buds weight, despite the fertilization season, compared with CK (Figure 3C). Compared with CK, the concentration of the total amino acids increased by 61% in TS, 14.6% in TW, and 87.6% in TWS (Figure 3D), whereby the concentration of the total polyphenols decreased by 3% in TS and 6.5% in TWS (Figure 3E). Among all the treatments, the polyphenols/amino acids ratio was the lowest in TWS (Figure 3F). Thus, the applications of foliar N two times at the winter dormancy stage and two times at the spring dormancy stage were chosen to investigate the effects of the different types of foliar N fertilizers as well as the effects of foliar N application on tea plants under reduced soil fertilization levels.

### Effects of Different Types of Foliar Fertilizers on Tea Plants

**FIGURE 4:**
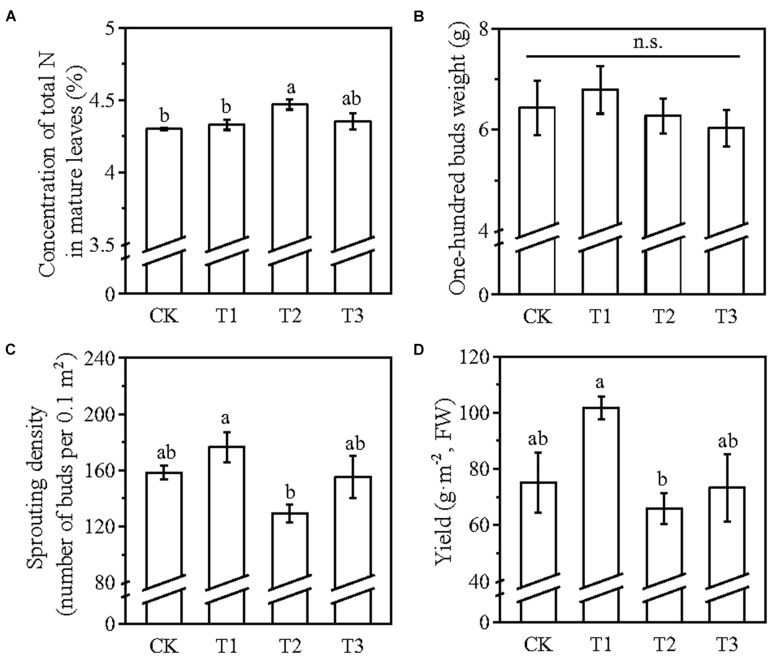
The effect of different types of foliar fertilizers on the total N concentrations of the mature leaves before the sprouting of dormant buds **(A)**, the 100 buds weight **(B)**, sprouting density **(C)**, and yield of spring tea **(D)**. FW, fresh weight. T1, foliar application of amino acid liquid fertilizer; T2, foliar application of 2% (w/v) urea; T3, foliar application of a commercially available auxin-containing fertilizer; CK, foliar application of water. Data presented mean ± SE; *n* = 4 in panel **(A)**, 6 in panel **(C)**, 3 in panel **(B,D)**. Lowercase letters indicated significant differences (*P* < 0.05; ANOVA, followed by the Duncan test). n.s., no significant difference.

**FIGURE 5:**
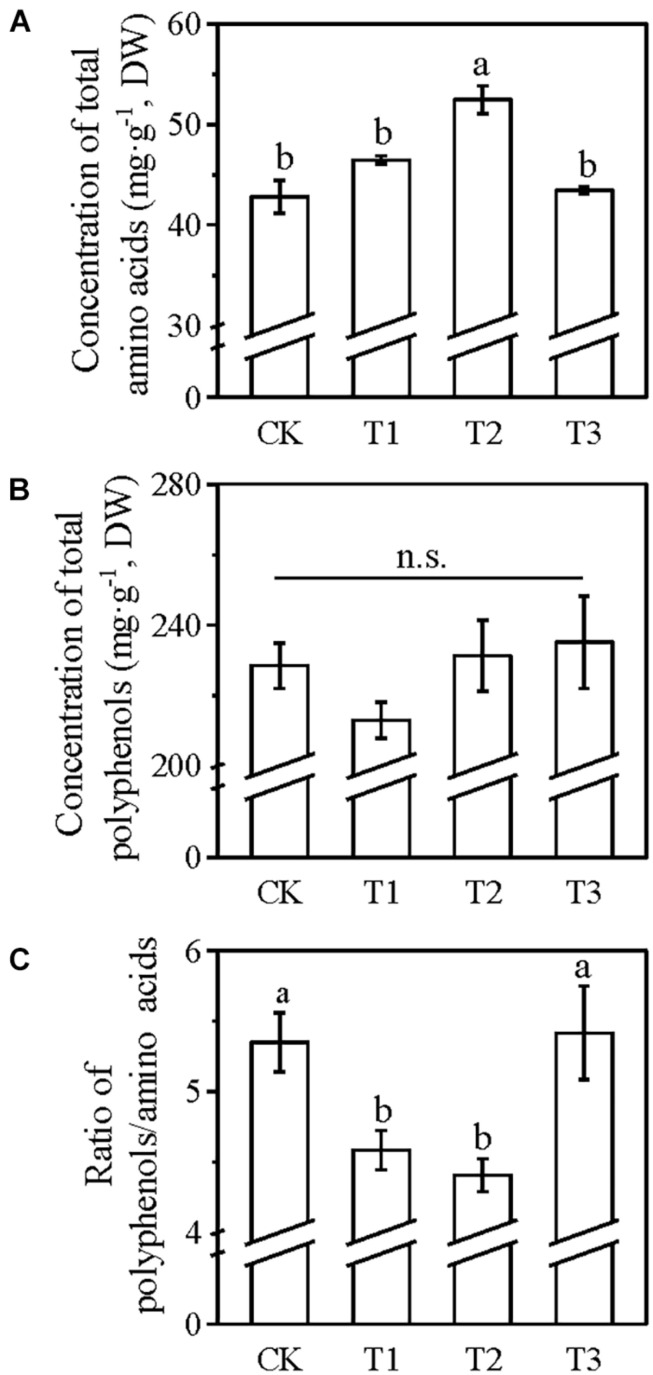
The effect of different types of foliar fertilizers on the concentrations of the total amino acids **(A)**, polyphenols **(B)**, and the polyphenol/amino acid ratio **(C)** of spring tea. DW. T1, foliar application of amino acid liquid fertilizer; T2, foliar application of 2% (w/v) urea; T3, foliar application of a commercially available auxin-containing fertilizer; CK, foliar application of water. Values were shown as means ± SE (*n* = 3). Lowercase letters indicated significant differences (*P* < 0.05; ANOVA, followed by the Duncan test). n.s., no significant difference.

Compared with CK, the foliar application of 2% urea (T2) significantly increased the total N concentration of the mature leaves (before the sprouting of dormant buds) by 3.9% in the field experiment, whereby the foliar application of amino acid liquid fertilizer (T1) and auxin-containing fertilizer (T3) increased the total N concentration of the mature leaves by 0.7 and 1.3%, respectively (Figure 4A). Compared with CK, the T1 treatment showed the highest 100 buds weight, while the T2 and T3 treatments reduced the 100 buds weight; and no significant difference was observed among all the treatments (Figure 4B). Among all the treatments, the sprouting density was the highest in T1 (14.9% higher than CK and significantly higher than T2) (Figure 4C). Only the T1 treatment increased the yield of spring tea (35.5% higher than CK and significantly higher than T2) (Figure 4D). The total amino acid concentration was the highest in T2, followed by T1, and T3 (22.5, 8.6, and 1.5% higher than CK, respectively) (Figure 5A). The concentration of the total polyphenols did not have a significant difference among the treatments (Figure 5B). The polyphenols/amino acids ratio of T1 and T2 were significantly lower than those of CK and T3, but there was no significant difference between T1 and T2 (Figure 5C). Together, these results suggested that the amino acid liquid fertilizer and the 2% urea liquid are better than the auxin-containing fertilizer for tea plants among the three fertilizers tested.

### Effect of Foliar N Application on Tea Plants Under a Reduced Soil Fertilization Level

**FIGURE 6:**
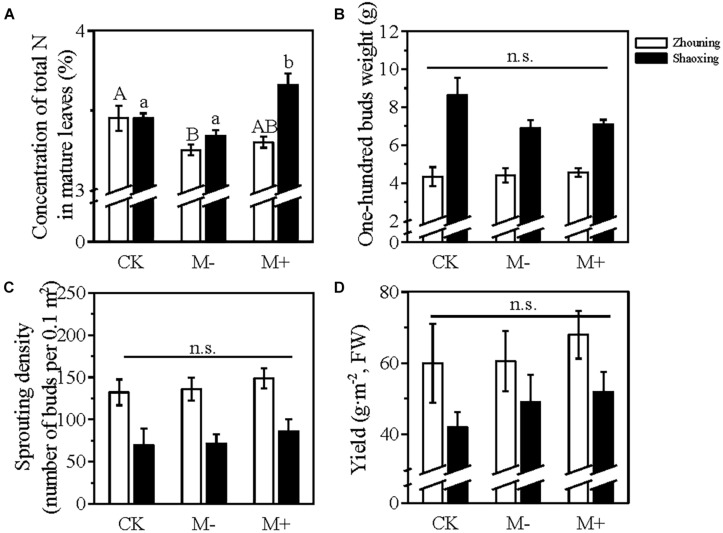
Field experiment investigating the effect of foliar N application on tea plants grown under a reduced soil fertilization level. **(A)** The concentration of total N in the mature leaves before the sprouting of dormant buds; **(B)** 100 buds weight; **(C)** sprouting density; **(D)** yield of spring tea. FW, fresh weight. CK, tea plants cultivated using the local soil fertilization level in Zhouning or Shaoxing without foliar N application; M− & M+, tea plants cultivated in the presence of 25% reduced soil fertilization level without (M−) or with (M+) foliar N application. Values were shown as means ± SEs [*n* = 6 for all the samples from Shaoxing, *n* = 4 for samples in panel **(A)** from Zhouning, *n* = 9 for samples in panels **(B–D)** from Zhouning]. The significance of different treatments from the same district was evaluated using one-way ANOVA followed by a Duncan test at a significance level of *P*_0_._05_ in SPSS 22.0 software. Statistically significant differences (*P* < 0.05) among the treatments in Zhouning and Shaoxing were shown with a capital letter and a lower case letter displayed above the bar plot, respectively; n.s. indicated no significance.

Compared with CK, the 25% reduced input of soil fertilizer (M−) decreased the total N concentration of the mature leaves in tea plantations in both Zhouning (decreased by 5.8% in M−) and Shaoxing (decreased by 3% in M−) (Figure 6A). However, the foliar application on the canopy of the tea plants complemented this reduction, with a 4% reduction in Zhouning (M+) and a 6% increment in Shaoxing (M+) (Figure 6A). The 100 buds weight among the treatments in the tea plantations did not show a significant difference (Figure 6B). Although the reduction of soil fertilizer reduced the 100 buds weight, the application of foliar fertilizer could complement this reduction (Figure 6B). After the reduced input of soil fertilizer, compared with CK, the sprouting density and yield of the spring tea in M− increased slightly in both Zhouning and Shaoxing; whereas this increment was further enhanced by the application of foliar fertilizer on the canopy of the tea plants in those two tea plantations (Figures 6C,D).

**FIGURE 7:**
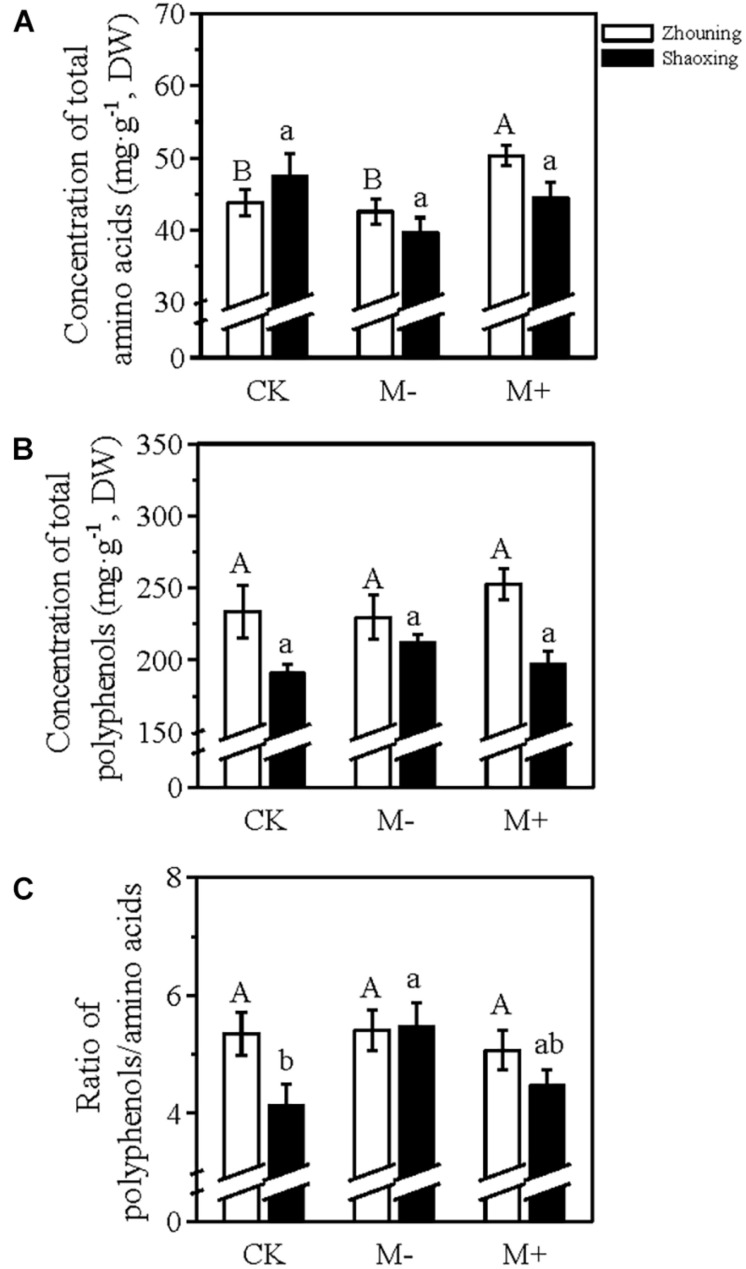
The changes of the total amino acids **(A)**, polyphenols **(B)**, and the polyphenol/amino acid ratio **(C)** of tea plants grown under a 25% reduction of local soil fertilization level with (M+) or without (−) foliar N application. DW, dry weight. CK, tea plants cultivated with the local soil fertilization level in Zhouning or Shaoxing but without foliar N application. Values were shown as means ± SEs (*n* = 6 for all the samples from Shaoxing and Zhouning). The significance of different treatments from the same district was evaluated using one-way ANOVA followed by a Duncan test at a significance level of *P*_0_._05_ in the SPSS 22.0 software. Statistically significant differences (*P* < 0.05) among the treatments in Zhouning and Shaoxing were shown with a capital letter and a lower case letter displayed above the bar plot, respectively.

The changes in the concentrations of the total amino acids and polyphenols were the same in both Zhouning and Shaoxing (Figures 7A,B). In Shaoxing, the concentration of the total amino acids in M− was lower than that in CK (Figure 7A), while the total polyphenols concentration in M− was higher than that in CK (Figure 7B), resulting in a significantly higher polyphenol/amino acid ratio in M− than in CK (Figure 7C). After the application of foliar N, the polyphenols/amino acids ratio in M+ was 5.59 in Zhouning (vs. 5.34 in CK) and 4.47 in Shaoxing (vs. 4.14 in CK), implying that the M+ treatment could somehow complement the quality of spring tea. These results suggested that the foliar N application on the canopy of the tea plants can be used as a strategy to complement the reduced input of soil fertilizer for the sustainable development of tea production.

### Confirmation of the Effect of Foliar N Application on Tea Plants Under a Reduced Soil Fertilization Manner Using the Foliar ^15^N-Urea as N Source

**FIGURE 8:**
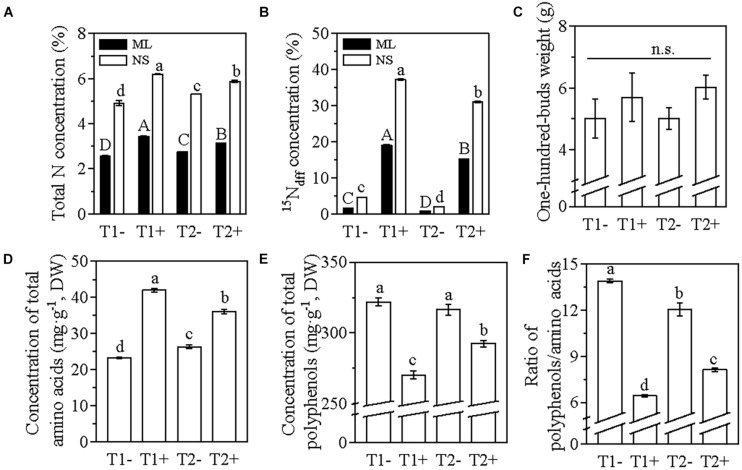
The ^15^N-labeling pot experiment investigating the effect of foliar N application on tea plants grown under a reduced soil N fertilization level. **(A)** The concentration of total N in mature leaves and new shoots (NS). **(B)** ^15^N derived from the ^15^N-urea in ML before the sprouting of dormant buds and in NS sprouted from the dormant buds. **(C–F)** Spring tea, one bud, and two leaves samples. T1, 25% reduced soil N level (1.5 g of pure N per pot) without (T1−) or with (T1+) foliar ^15^N-urea application; T2, local soil N fertilization level (2 g of pure N per pot) without (T2−) or with (T2+) foliar ^15^N-urea application. DW, dry weight. Data represented mean ± SE; *n* = 3 for samples in panels **(A,B,F)**; *n* = 5 for samples in panel **(C)**; *n* = 9 for samples in panel **(D)**; *n* = 6 for samples in panel **(E)**. Lowercase letters indicated significant differences of NS, while capital letters indicated the significance of ML (*P* < 0.05; ANOVA, followed by the Duncan test).

The total N concentration of the mature leaves and new shoots in T1− was significantly lower than that in T2−, whereas the total N concentration of the mature leaves and new shoots in T1 + was significantly higher than that in T2+ (Figure 8A). The abundance of ^15^N in the mature leaves (N_dff_ = 19%) and new shoots (N_dff_ = 37%) of the tea plants in T1 + were significantly higher than that in T2 + (N_dff_ = 12.19% in the mature leaves and 31% in the new shoots) (Figure 8B). These results indicate that the reduced input of soil N fertilizer promotes the absorption of foliar N in tea plants. The foliar N application increased the 100 buds weight in both T1+ and T2+; the 100 buds weight in T2+ was slightly, but not significantly, higher than that in T1+ (Figure 8C). In T1−, the reduced input of soil N fertilizer deteriorated the quality of spring tea, resulting in the significantly lowest concentration of total amino acids and the highest concentration of total polyphenols, and consequently the highest polyphenols/amino acids ratio (Figures 8D–F). Interestingly, after the application of foliar N in T1+, the changes in the concentration of the total amino acids and polyphenols were contrary to that observed in T1−, with a significantly higher amino acid concentration and lower polyphenols concentration and polyphenol/amino acid ratio (Figures 8D–F). The quality components of spring tea in T1+ were the most superior, followed by those in T2+, T2−, and T1− (Figures 8D–F). Hence, the application of foliar N on the canopy of the tea plants is an effective strategy for reducing the input of soil fertilizer, thus facilitating sustainable tea production.

## Discussion

Foliar fertilizer application has been proven to be an excellent method for supplying nutrients to plants. The purpose of foliar fertilization is not to replace soil fertilization but to supplement the nutrients needed by plants at short or critical growth stages. Nowadays, in China, <50% of the fertilizer applied to fields goes to the crops for which it was intended and much of the rest leaches into the environment (Zhang et al., 2013). More than 30% of the Chinese tea plantations are in excess of fertilizers, and a drastic reduction in the amount of fertilizer (especially for N fertilizer) applied to tea fields is urgently required (Ni et al., 2019). However, a marked decline in the leaf N concentration should be avoided under low fertilizer conditions to maintain the quality of tea (Okano and Matsuo, 1996). Improving the fertilizer application technique is one of the most promising approaches for achieving these contradictory purposes. Thus, in this study, we explored whether foliar N application could complement the reduction in the soil fertilizer input in tea plantations since the nutrients are directly delivered to the sink organs.

In tea plants, foliar-applied N is quickly absorbed within the first 6 h, and absorption is accomplished within 2–3 days after the application of foliar urea (Ruan and Gerendas, 2015). The N absorbed through the roots during the dormancy period is stored in the tea plants and then remobilized to support the next growth phase (Liu et al., 2017; Fan et al., 2019, 2020; Ma et al., 2019). The remobilization dynamics of the storage of N can affect the quality of tea in the spring season (Liu et al., 2016). Moreover, foliar N application usually occurs in spring to promote the sprouting of dormant buds or during the harvest time to increase the yield of spring tea. Good quality spring tea has a low polyphenol/amino acid ratio. Our results showed that foliar applications of N two times at the winter dormancy stage were sufficient for increasing the concentration of N in the mature leaves before the sprouting of dormant buds and also for improving the quality and yield of spring tea (Figures 1, 2). The combined application of foliar N at both winter and spring dormancy stages was the best method of foliar fertilizer application (Figure 3), without causing the accumulation of any chemical residues on the spring tea.

The foliar spray of urea solution has been widely applied in agriculture to improve the quality and yield of field and horticultural crops (Klein and Weinbaum, 1985; Rahman et al., 2014). The absorbed urea is hydrolyzed in the cytosols by the urease, although the urea persisted for 36–72 h in plant leaves after foliar spray (Bowman and Paul, 1990; Witte et al., 2002). The uptake of amino acids by plants is energetically more advantageous than the absorption of urea in the soil because the plant does not need the energy to assimilate the absorbed N and later incorporate it into amino acids (Jones and Kielland, 2002). The amino acids applied to crops are usually associated with products based on algae extracts or fermented animal or vegetable wastes. The amino acid liquid fertilizer used in this study was a hydrolyzed product of animal carcasses (Liu et al., 2016; Wang et al., 2019). Among the three foliar fertilizers tested in this study, the amino acid liquid fertilizer showed the best performance, as evident from the increase in the 100 buds weight, sprouting density, and yield of spring tea compared with the other two foliar fertilizers, while the foliar application of urea caused a higher increment in the N concentration of the mature leaves beneath the dormant buds (Figure 4). The application of both urea and amino acid improved the quality of the spring tea, with no significant difference between these two treatments (Figure 5). Spring tea has higher economic value than summer and autumn teas (Liu et al., 2016). Therefore, farmers use large volumes of auxin-containing foliar fertilizers to promote the sprouting of dormant buds and increase the yield of spring tea. However, foliar application of auxin-containing fertilizer deteriorated the quality of spring tea and had a negative impact on other factors related to the yield of spring tea (Figures 4, 5). Our results suggest that tea plantation farmers should not choose auxin-containing fertilizers as foliar fertilizers.

The tea plant is an evergreen plant species, and the young shoots of tea plants are generally used for the production of tea products. The annual pruning of tea plants causes a substantial loss of N. The N status of tea plants determines the total concentrations of free amino acids, polyphenols, and caffeine (Ruan et al., 2010). Hence, the N fertilization in tea plantations is vital for the growth and development of tea plants. To establish sustainable tea production, studies suggest that the input of soil fertilizer in tea plantations should be reduced to 25% of the local soil fertilization level (Ni et al., 2019). In this study, we applied foliar N fertilizer to tea plants during their dormancy stage under a 25% reduced soil fertilization level. Although the reduced input of soil fertilizer decreased the total concentration of N in the mature leaves before the sprouting of dormant buds, the 100 buds weight, sprouting density, and yield of spring tea were not significantly affected (Figure 6). This suggests that the soil fertilization level in these two tea plantations located in different provinces of China was in excess. Only 7.5 L/ha were applied for one single spraying of the liquid amino acid fertilizer, providing just around 310 g N/ha. The application of this foliar N on the canopy of the tea plants not only increased the total concentration of N in the mature leaves but also improved the factors related to the yield of spring tea (Figure 6), indicating an effective N utilization. This was confirmed by the pot experiment in which ^15^N-urea was used as the source of foliar N (Figures 8A,B). The changes in the concentrations of total amino acids and polyphenols and the polyphenols/amino acids ratio showed that the quality of the spring tea harvested from the tea plants grown under the reduced fertilization pattern was lower than that harvested from the tea plants grown under the local fertilization level; however, the foliar application could somehow recover the quality of spring tea (Figures 7, 8). These results suggested that the decreased total N concentration in the mature leaves and the deteriorated quality of spring tea caused by the reduction of soil fertilization level could be improved through foliar N application although containing a relatively lower N concentration compared with the omission of the soil fertilization levels.

## Conclusion

In this study, the applications of foliar N two times at the winter dormancy stage significantly increased the N concentration of mature leaves before the sprouting of dormant buds and improved the quality of spring tea. The combined application of foliar N at the winter dormancy stage and at the spring dormancy stage was the most effective method of foliar N application in tea plantations. This strategy could be used to reduce the soil fertilization level in tea plantations, without decreasing the total N concentration of the mature leaves or deteriorating the quality of spring tea.

## Data Availability Statement

The original contributions presented in the study are included in the article/supplementary material, further inquiries can be directed to the corresponding author.

## Author Contributions

M-YL and QZ conceived and designed the experiments and analyzed the data. M-YL, DT, LM, YS, and QZ performed the experiments. M-YL wrote the manuscript. QZ and JR finalized the manuscript. All authors read and approved the final manuscript.

## Conflict of Interest

The authors declare that the research was conducted in the absence of any commercial or financial relationships that could be construed as a potential conflict of interest.

## Publisher’s Note

All claims expressed in this article are solely those of the authors and do not necessarily represent those of their affiliated organizations, or those of the publisher, the editors and the reviewers. Any product that may be evaluated in this article, or claim that may be made by its manufacturer, is not guaranteed or endorsed by the publisher.

## Footnotes

1
https://jylyswkj.en.made-in-china.com/

2
http://lvyanghuagong.3456.tv/
